# Supplementary figures and images for: Adipocyte-specific deletion of Dbc1 does not recapitulate healthy obesity phenotype but suggests regulation of inflammation signaling
Source: PLoS One. 2025 May 2;20(5):e0322732. doi: 10.1371/journal.pone.0322732 (PMC12048159; doi:10.1371/journal.pone.0322732)

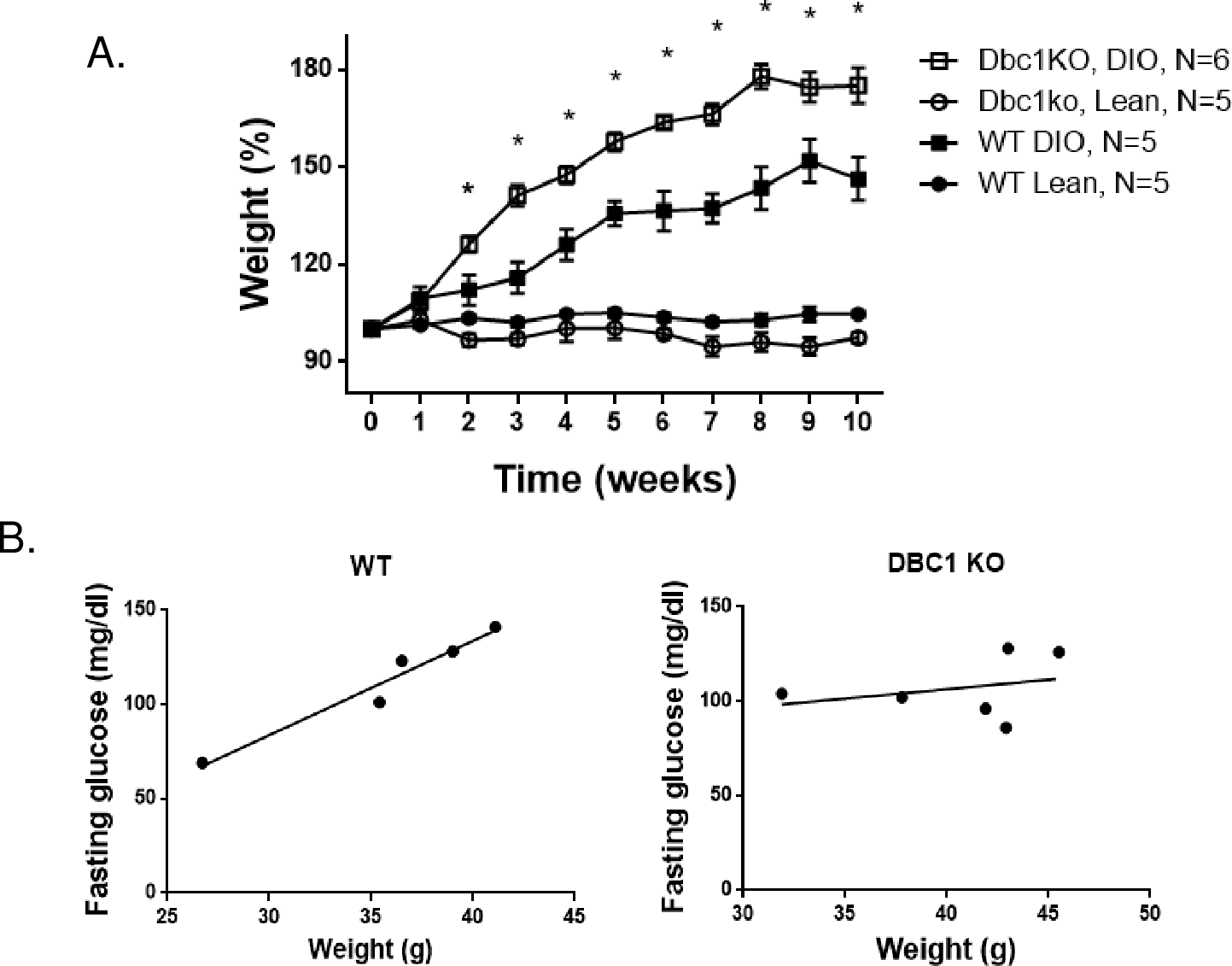

Supplement: S1 File — Testing of gRNA Efficiency in Inducible 3T3-Cas9 Cells. (A) Western blot analysis of total cell extracts at various time points following incubation with doxycycline. We generated a 3T3-L1 cell line where Cas9 expression is controlled by doxycycline. (B) Immunofluorescence images of cells after 48 hours of incubation with doxycycline. Nuclei are stained with DAPI, and Flag-Cas9 is detected by immunofluorescence following doxycycline induction. Bar = 50 µm. (C) Acrylamide gel electrophoresis stained with EtBr showing PCR products using genomic DNA from 3T3-Cas9 cells after transfection with various gRNAs and treatment with doxycycline. Black arrows indicate the formation of heteroduplexes, which are absent in the controls of uninduced and untransfected cells, as well as in induced but untransfected cells (Ctrl and Ctrl2, respectively). A noticeable decrease in amplicon yield suggests significant modifications following Cas9 cleavage and DNA repair processes. (D) Western blot analysis of DBC1 protein levels in the stromal vascular fraction of adipose tissue from Dbc1LoxP/LoxP and Dbc1LoxP/LoxP;CRE mice, showing DBC1 and Tubulin as a loading control. (E) Densitometric analysis of Dbc1 normalized to Tubulin and expressed as fold change relative to Dbc1LoxP/LoxP. Data are presented as mean ± SD (n = 4 per group). Statistical significance was determined using an unpaired t-test. Fig S2. Metabolic protection of Dbc1 KO obese mice. (A) Weight gain in WT and Dbc1 KO mice fed a high-fat diet on a C57BL/6J background, showing increased weight gain in Dbc1 KO mice compared to WT control mice. (B) Fasting glucose vs. body weight in obese mice shows a positive correlation between fasting glucose and body weight in WT (wild-type) animals, as expected. However, dbc1 knockout (KO) animals do not show this correlation, reflecting the protection against developing metabolic syndrome observed in Dbc1 KO animals. Fig S3. Pathway Analysis of Gene Expression Changes in Dbc1 KO adipocytes. [file pone.0322732.s001.zip › PACE Corrected/S2.tif]

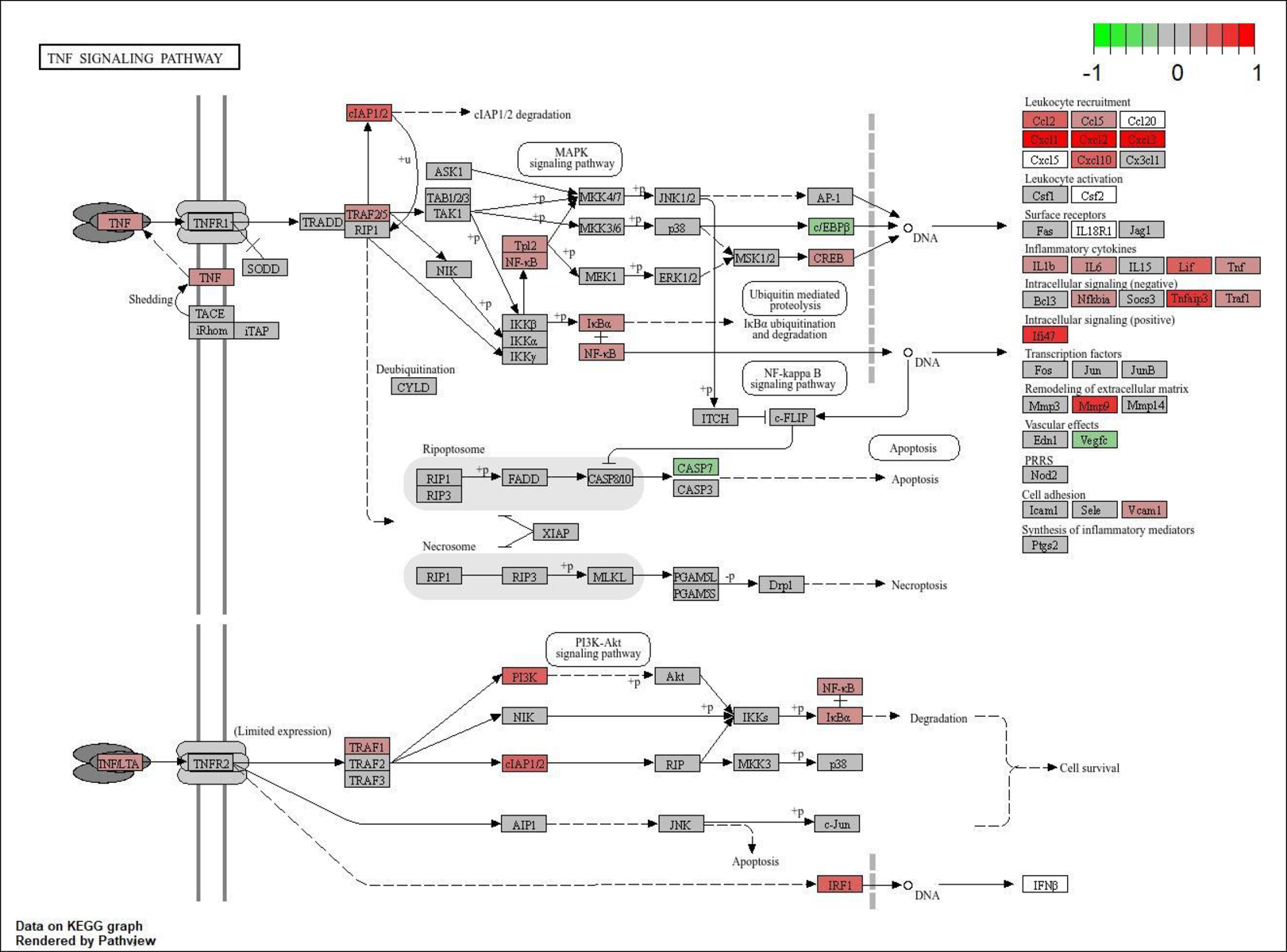

Supplement: S1 File — Testing of gRNA Efficiency in Inducible 3T3-Cas9 Cells. (A) Western blot analysis of total cell extracts at various time points following incubation with doxycycline. We generated a 3T3-L1 cell line where Cas9 expression is controlled by doxycycline. (B) Immunofluorescence images of cells after 48 hours of incubation with doxycycline. Nuclei are stained with DAPI, and Flag-Cas9 is detected by immunofluorescence following doxycycline induction. Bar = 50 µm. (C) Acrylamide gel electrophoresis stained with EtBr showing PCR products using genomic DNA from 3T3-Cas9 cells after transfection with various gRNAs and treatment with doxycycline. Black arrows indicate the formation of heteroduplexes, which are absent in the controls of uninduced and untransfected cells, as well as in induced but untransfected cells (Ctrl and Ctrl2, respectively). A noticeable decrease in amplicon yield suggests significant modifications following Cas9 cleavage and DNA repair processes. (D) Western blot analysis of DBC1 protein levels in the stromal vascular fraction of adipose tissue from Dbc1LoxP/LoxP and Dbc1LoxP/LoxP;CRE mice, showing DBC1 and Tubulin as a loading control. (E) Densitometric analysis of Dbc1 normalized to Tubulin and expressed as fold change relative to Dbc1LoxP/LoxP. Data are presented as mean ± SD (n = 4 per group). Statistical significance was determined using an unpaired t-test. Fig S2. Metabolic protection of Dbc1 KO obese mice. (A) Weight gain in WT and Dbc1 KO mice fed a high-fat diet on a C57BL/6J background, showing increased weight gain in Dbc1 KO mice compared to WT control mice. (B) Fasting glucose vs. body weight in obese mice shows a positive correlation between fasting glucose and body weight in WT (wild-type) animals, as expected. However, dbc1 knockout (KO) animals do not show this correlation, reflecting the protection against developing metabolic syndrome observed in Dbc1 KO animals. Fig S3. Pathway Analysis of Gene Expression Changes in Dbc1 KO adipocytes. [file pone.0322732.s001.zip › PACE Corrected/S3.tif]

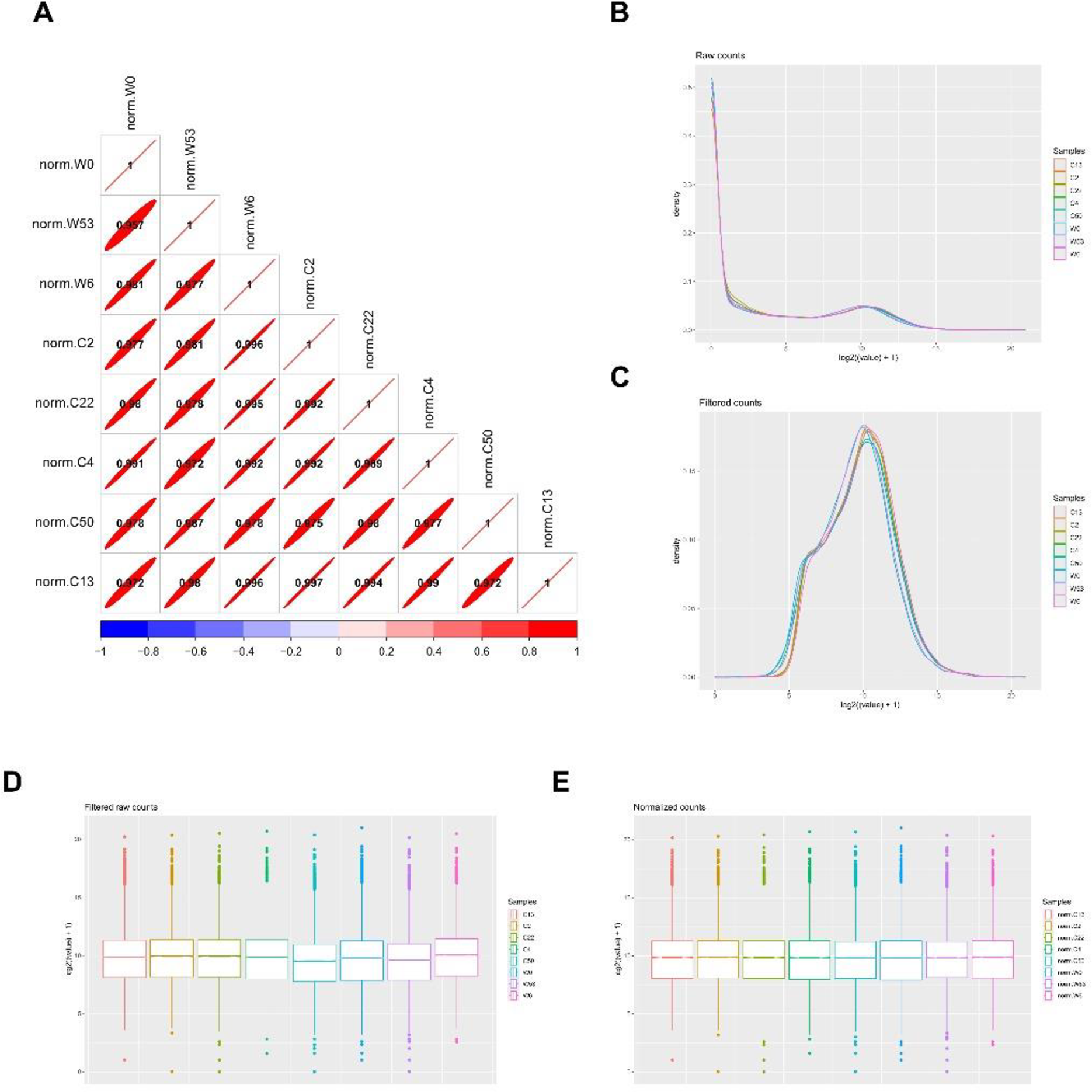

Supplement: S1 File — Testing of gRNA Efficiency in Inducible 3T3-Cas9 Cells. (A) Western blot analysis of total cell extracts at various time points following incubation with doxycycline. We generated a 3T3-L1 cell line where Cas9 expression is controlled by doxycycline. (B) Immunofluorescence images of cells after 48 hours of incubation with doxycycline. Nuclei are stained with DAPI, and Flag-Cas9 is detected by immunofluorescence following doxycycline induction. Bar = 50 µm. (C) Acrylamide gel electrophoresis stained with EtBr showing PCR products using genomic DNA from 3T3-Cas9 cells after transfection with various gRNAs and treatment with doxycycline. Black arrows indicate the formation of heteroduplexes, which are absent in the controls of uninduced and untransfected cells, as well as in induced but untransfected cells (Ctrl and Ctrl2, respectively). A noticeable decrease in amplicon yield suggests significant modifications following Cas9 cleavage and DNA repair processes. (D) Western blot analysis of DBC1 protein levels in the stromal vascular fraction of adipose tissue from Dbc1LoxP/LoxP and Dbc1LoxP/LoxP;CRE mice, showing DBC1 and Tubulin as a loading control. (E) Densitometric analysis of Dbc1 normalized to Tubulin and expressed as fold change relative to Dbc1LoxP/LoxP. Data are presented as mean ± SD (n = 4 per group). Statistical significance was determined using an unpaired t-test. Fig S2. Metabolic protection of Dbc1 KO obese mice. (A) Weight gain in WT and Dbc1 KO mice fed a high-fat diet on a C57BL/6J background, showing increased weight gain in Dbc1 KO mice compared to WT control mice. (B) Fasting glucose vs. body weight in obese mice shows a positive correlation between fasting glucose and body weight in WT (wild-type) animals, as expected. However, dbc1 knockout (KO) animals do not show this correlation, reflecting the protection against developing metabolic syndrome observed in Dbc1 KO animals. Fig S3. Pathway Analysis of Gene Expression Changes in Dbc1 KO adipocytes. [file pone.0322732.s001.zip › PACE Corrected/S4.tif]

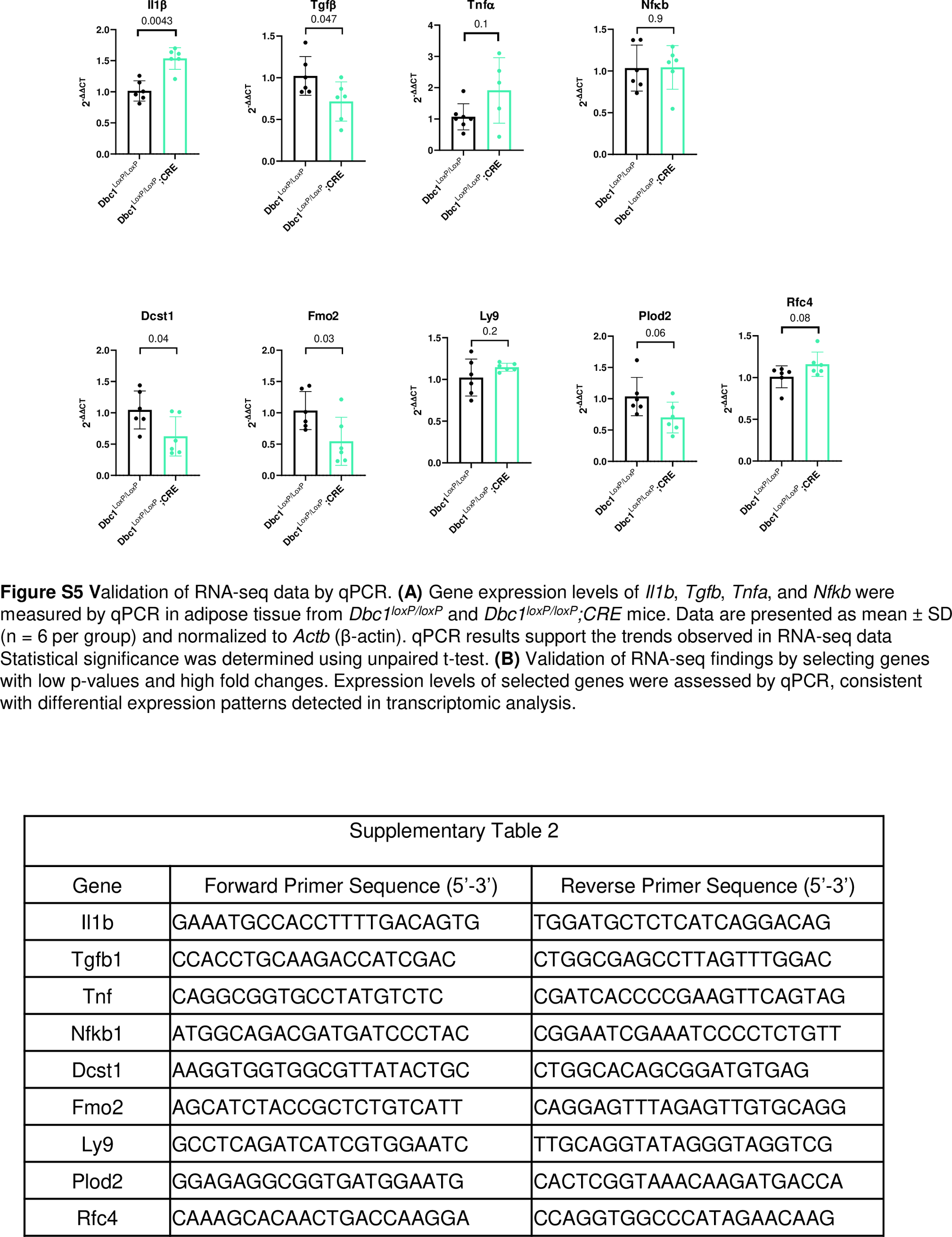

Supplement: S1 File — Testing of gRNA Efficiency in Inducible 3T3-Cas9 Cells. (A) Western blot analysis of total cell extracts at various time points following incubation with doxycycline. We generated a 3T3-L1 cell line where Cas9 expression is controlled by doxycycline. (B) Immunofluorescence images of cells after 48 hours of incubation with doxycycline. Nuclei are stained with DAPI, and Flag-Cas9 is detected by immunofluorescence following doxycycline induction. Bar = 50 µm. (C) Acrylamide gel electrophoresis stained with EtBr showing PCR products using genomic DNA from 3T3-Cas9 cells after transfection with various gRNAs and treatment with doxycycline. Black arrows indicate the formation of heteroduplexes, which are absent in the controls of uninduced and untransfected cells, as well as in induced but untransfected cells (Ctrl and Ctrl2, respectively). A noticeable decrease in amplicon yield suggests significant modifications following Cas9 cleavage and DNA repair processes. (D) Western blot analysis of DBC1 protein levels in the stromal vascular fraction of adipose tissue from Dbc1LoxP/LoxP and Dbc1LoxP/LoxP;CRE mice, showing DBC1 and Tubulin as a loading control. (E) Densitometric analysis of Dbc1 normalized to Tubulin and expressed as fold change relative to Dbc1LoxP/LoxP. Data are presented as mean ± SD (n = 4 per group). Statistical significance was determined using an unpaired t-test. Fig S2. Metabolic protection of Dbc1 KO obese mice. (A) Weight gain in WT and Dbc1 KO mice fed a high-fat diet on a C57BL/6J background, showing increased weight gain in Dbc1 KO mice compared to WT control mice. (B) Fasting glucose vs. body weight in obese mice shows a positive correlation between fasting glucose and body weight in WT (wild-type) animals, as expected. However, dbc1 knockout (KO) animals do not show this correlation, reflecting the protection against developing metabolic syndrome observed in Dbc1 KO animals. Fig S3. Pathway Analysis of Gene Expression Changes in Dbc1 KO adipocytes. [file pone.0322732.s001.zip › PACE Corrected/S5.tif]

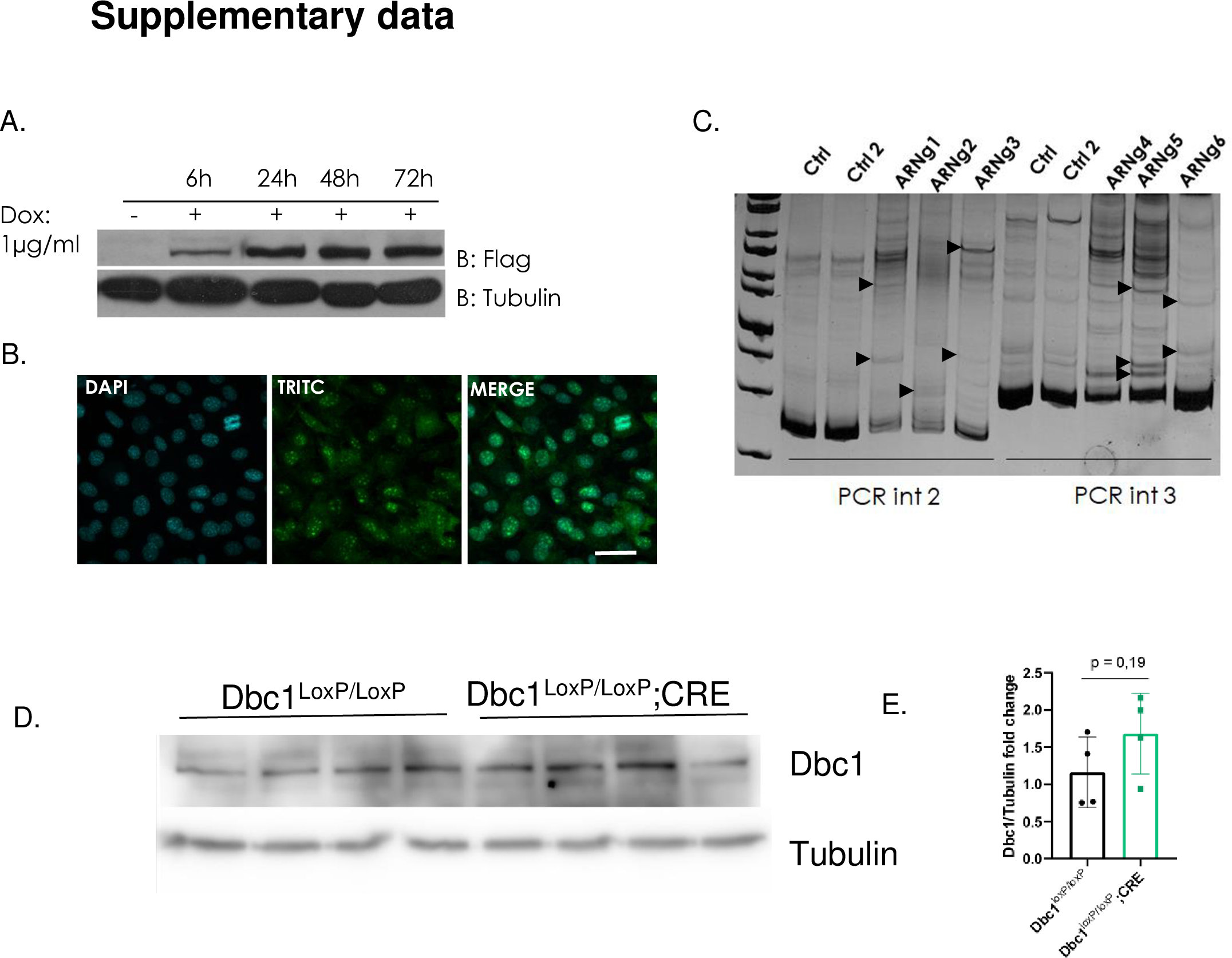

Supplement: S1 File — Testing of gRNA Efficiency in Inducible 3T3-Cas9 Cells. (A) Western blot analysis of total cell extracts at various time points following incubation with doxycycline. We generated a 3T3-L1 cell line where Cas9 expression is controlled by doxycycline. (B) Immunofluorescence images of cells after 48 hours of incubation with doxycycline. Nuclei are stained with DAPI, and Flag-Cas9 is detected by immunofluorescence following doxycycline induction. Bar = 50 µm. (C) Acrylamide gel electrophoresis stained with EtBr showing PCR products using genomic DNA from 3T3-Cas9 cells after transfection with various gRNAs and treatment with doxycycline. Black arrows indicate the formation of heteroduplexes, which are absent in the controls of uninduced and untransfected cells, as well as in induced but untransfected cells (Ctrl and Ctrl2, respectively). A noticeable decrease in amplicon yield suggests significant modifications following Cas9 cleavage and DNA repair processes. (D) Western blot analysis of DBC1 protein levels in the stromal vascular fraction of adipose tissue from Dbc1LoxP/LoxP and Dbc1LoxP/LoxP;CRE mice, showing DBC1 and Tubulin as a loading control. (E) Densitometric analysis of Dbc1 normalized to Tubulin and expressed as fold change relative to Dbc1LoxP/LoxP. Data are presented as mean ± SD (n = 4 per group). Statistical significance was determined using an unpaired t-test. Fig S2. Metabolic protection of Dbc1 KO obese mice. (A) Weight gain in WT and Dbc1 KO mice fed a high-fat diet on a C57BL/6J background, showing increased weight gain in Dbc1 KO mice compared to WT control mice. (B) Fasting glucose vs. body weight in obese mice shows a positive correlation between fasting glucose and body weight in WT (wild-type) animals, as expected. However, dbc1 knockout (KO) animals do not show this correlation, reflecting the protection against developing metabolic syndrome observed in Dbc1 KO animals. Fig S3. Pathway Analysis of Gene Expression Changes in Dbc1 KO adipocytes. [file pone.0322732.s001.zip › PACE Corrected/S1.tif]
